# Advancing glioblastoma treatment by targeting metabolism

**DOI:** 10.1016/j.neo.2024.100985

**Published:** 2024-03-12

**Authors:** Jinyi Zhao, Xuemei Ma, Peixian Gao, Xueqi Han, Pengxiang Zhao, Fei Xie, Mengyu Liu

**Affiliations:** a College of Chemistry and Life Science, Beijing University of Technology, Beijing, China; b Beijing Molecular Hydrogen Research Center, Beijing, China

**Keywords:** Glioblastoma, Metabolism, Signaling pathway, Therapy

## Abstract

Alterations in cellular metabolism are important hallmarks of glioblastoma(GBM). Metabolic reprogramming is a critical feature as it meets the higher nutritional demand of tumor cells, including proliferation, growth, and survival. Many genes, proteins, and metabolites associated with GBM metabolism reprogramming have been found to be aberrantly expressed, which may provide potential targets for cancer treatment. Therefore, it is becoming increasingly important to explore the role of internal and external factors in metabolic regulation in order to identify more precise therapeutic targets and diagnostic markers for GBM. In this review, we define the metabolic characteristics of GBM, investigate metabolic specificities such as targetable vulnerabilities and therapeutic resistance, as well as present current efforts to target GBM metabolism to improve the standard of care.

## Introduction

GBM is one of the most common primary malignancies of the central nervous system [1], [2], [3]. This particular type of glioma is highly lethal, with a notably poor prognosis and a median survival rate of merely 12.1 months. GBM have a modified metabolism to support a variety of bioenergetic and biosynthetic needs for tumor development, invasion, and treatment resistance [4]. In GBMs, alterations involve to multiple metabolism pathways, such as oxidative phosphorylation (OXPHOS), pentose phosphate pathway (PPP), fatty acid biosynthesis and oxidation, and nucleic acid biosynthesis. All of these changed pathways are implicated in the increased growth of tumors. Moreover, activation of PI3K/Akt/mTOR signaling, increased glycolysis flow and lipid storage all have an effect on the metabolic processes in glioblastoma. Despite the identification of changes in signaling pathways within targetable core pathways in GBM through recent studies [5,6], only poor therapeutic outcomes have been seen with medicines targeting particular molecular alterations [7]. This is partly explained by the challenge of administering physiologically active drug concentrations to the tumor [8], the existence of considerable signaling pathway redundancy [9], and the cellular heterogeneity of GBM [10]. The development of new efficient GBM treatment strategies continues to be extremely challenge.

## Metabolic features of glioblastoma

### Glycolysis

In GBM, glycolysis plays an important role in tumor development, invasion, angiogenesis, and resistance to chemotherapy and radiation. Additionally, glycolysis shapes the tumor microenvironment (TME), which also regulates inflammatory and immunological responses [11], [12], [13], [14]. Studies show that reversing the energy production pathway back to OXPHOS could induce the differentiation of glioblastoma into astrocytes [15]. The brain employs alternate energy substrates like lactic acid and ketone bodies to sustain normal function under harsh environmental circumstances. Recently, an analysis focused on the gene expression profile of glycolysis and mitochondrial metabolism in brain tumor samples from lower-grade glioma and GBM patients, which showed that the gene expression of glycolytic enzymes is up-regulated in GBM samples [16]. The glycolysis and TCA involve several key enzymes that serve a vital purpose in GBM metabolism. The data showed that rate-limiting glycolytic enzyme hexokinase2(HK2) and pyruvate kinase M2(PKM2) were significantly increased in GBM patients which also have a higher correlation with development GBM. Another study determined that increased HK2 promotes tumor growth and resistance to apoptosis of cancer cells [17]. Furthermore, Zhimin Lu's et.al found HK2 activated the NF-κB pathway thus promoting PD-L1 expression and tumor immune escape by promoting phosphorylation and degradation of IκBα [18].

Phosphofructokinase 1(PFK1) is a downstream kinase of HK2 which plays as a critical mediator in glycolysis. Fructose 6 phosphate is isomerized from glucose to fructose 1,6-bisphosphate by phosphorylated HK2, which is then further phosphorylated by PFK1. In human GBM cells, the expression of PFK1 platelet isoform (PFKP) is closely related to PFK1 activity. Overexpressed PFKP and hyperactive glycolysis promote tumor growth by AKT activation in GBM specimens [19]. PKM2, besides being a significant enzyme in cancer metabolism, also facilitates cell proliferation, cancer cell invasion, and tumor formation through a non-metabolic mechanism. Yang et al found that activation of epidermal growth factor receptor (EGFR) induced PKM2 translocation to nuclear which accelerated tumor formation by increasing the expression of cyclin D1 in GBM U87 cell line and other human cancer cells [20], [21], [22], [23]. The activity of PKM2 is regulated by several mechanisms in GBM. Fructose-1,6-bisphosphate, which is the product of PFK1, is a potent allosteric activator of PKM2 [24]. PKM2 is also a target of tumor-suppressive miRNA-326 and maintains glioma stem cells [22]. Necrosis is a common feature of malignant tumors that corresponds to rapid tumor cell proliferation. Moreover, PKM2 is a regulator that allows cancer cells to adapt to this typical microenvironment through altered metabolism. Mitochondrial serine hydroxy methyltransferase (SHMT2) decreased PKM2 and reduces oxygen consumption to support cancer cell survival in ischemic zones of gliomas [25]. Additionally, as compared to healthy individuals, the majority gene expression of glycolytic, such as ALDOC, GAPDH, PGK1, and PGAM1, were considerably higher in GBM patients [3]. GAPDH has been demonstrated to enhance the survival of cancer cells and accelerate autophagy in gliomas [26]. According to Zhang et al., resident tissue macrophages produce IL-6 to stimulate PDPK1-dependent PGK1 phosphorylation in tumor cells, which in turn promotes tumor cell glycolysis and carcinogenesis. This mechanism regulates the progression of a PGK1-catalyzed process. Furthermore, analysis of data from GBM patients reveals a correlation between PGK1 phosphorylation, malignant grades, and prognosis of GBM patients [27]. In comparison to normal individuals, the expression of LDHA and GPI was significantly lower in GBM samples. Targeting key components of glycolysis, such as hexokinase 2 (HK2) and lactate dehydrogenase A (LDHA), with inhibitors could potentially selectively eliminate cancer cells [28]. Glycolysis can also be regulated with other metabolism pathways. Recent studies have demonstrated that the fructolysis mechanism, unique to the brain, plays a role in the Warburg effect. Specifically, it decreases mitochondrial respiration and aerobic glycolysis while enhancing OXPHOS. This effect may potentially contribute to metastasis under conditions of low oxygen availability.

### Tricarboxylic acid cycle

The TCA cycle is responsible for generating energy in the form of ATP through the oxidation of carbohydrates, fats, and proteins. It also provides building blocks for the synthesis of various molecules needed for cell growth and division. In cancer cells, alterations in metabolic pathways, including the TCA cycle, lead to changes in cellular metabolism that support the high energy demands of cancer cells and support their survival and proliferation. The pyruvate dehydrogenase (PDH) complex is an enzyme complex located within the mitochondrial matrix. It plays a crucial role in oxidative metabolism by irreversibly converting pyruvate into acetyl-CoA. PDH was phosphorylated and inactivated by Pyruvate dehydrogenase kinase (PDK), which results in reduced pyruvate oxidation in mitochondria and increased lactate synthesis in the cytosol. Prabhu et al., found that the activity of PDH is increased in the presence of Ras-mediated PDH phosphatase (PDP) expression. However, they also observed that this expression is suppressed in GBM patients. Interestingly, when PDP1 was restored, it resulted in a deceleration of GBM tumor development [29]. Acetyl-CoA, an essential metabolite in TCA cycle, undergoes oxidation to produce CO_2_ along with the generation of energy. This energy is initially stored in the form of NADH and FADH2. These coenzymes are subsequently oxidized, releasing protons and electrons that are utilized in ATP synthesis through OXPHOS [30]. Consequently, targeting OXPHOS represents a potential strategy for combating tumor cells. Studies have demonstrated that AG311 and Gboxin, both OXPHOS inhibitors, have the ability to inhibit tumor growth in glioblastoma (GBM). These compounds target the OXPHOS pathway, disrupting the production of ATP and ultimately impairing the energy metabolism of tumor cells. The inhibition of OXPHOS by AG311 and Gboxin has shown promising results in suppressing GBM tumor growth. Notably, α-ketoglutarate (α-KG) serves not only as a substrate for the production of CO_2_ and intermediate metabolites in the TCA cycle but also as a carbon backbone donor for the synthesis of amino acids such as aspartate and glutamate. In the context of GBM cells, it has been observed that these cells can replenish the TCA cycle by taking up aspartate and glutamate from the extracellular environment, which allows them to produce α-KG. This mechanism enables GBM cells to sustain the TCA cycle and maintain their metabolic activity [31]. Indeed, GBM cells primarily rely on glycolysis rather than the TCA cycle to produce energy for tumorigenesis. This metabolic preference, known as the Warburg effect, allows GBM to generate ATP through glycolysis, even in the presence of oxygen. Moreover, GBM cells modify the intermediates of the TCA cycle to meet the biosynthetic demands necessary for tumor growth and invasion. These modifications involve redirecting carbon flux toward the synthesis of amino acids, lipids, and nucleotides, which are essential for the rapid proliferation and metastases of GBM.

### The pentose phosphate pathway

The pentose phosphate pathway (PPP) is the initial branch of glycolysis involved in lipid biosynthesis, as well as the synthesis of nicotinamide adenine dinucleotide phosphate (NADPH) and nucleotides. In many cancers, the expression of PPP-associated proteins is up-regulated to support the synthesis of nucleotides for DNA repair and replication, as well as the production of NADPH for antioxidant defense mechanisms [32]. Higher expression of de novo pyrimidine synthesis enzymes and genes has been found to be associated with a poor prognosis in GBM patients, indicating that the abnormal PPP may impact the progression of GBM [33]. Some of the key intermediates of PPP include glucose-6-phosphate (G6P), 6-phosphogluconolactone (6PGL), 6-phosphogluconate (6PG), ribulose-5-phosphate (Ru5P), and ribose-5-phosphate (R5P) play critical roles in various cellular processes. Changes in the levels of these intermediate metabolites and their associated enzymes are also closely associated with the development of GBM. In comparison to healthy individuals, GBM patients exhibit significantly elevated levels of the enzymes 6-phosphogluconolactonase (PGLS) and 6-phosphogluconate dehydrogenase (PGD). However, the expression of glucose 6-phosphate dehydrogenase (G6PD) is downregulated in GBM patients. Furthermore, the increased levels of PGLS and PGD promote the production of R5P and NADPH, which are essential for nucleotide synthesis and energy production, supporting the proliferation of tumor cells. In recent studies, it has been demonstrated that STAT3 serves as a novel enhancer of phosphoinositide 3-kinase-activating Akt (PIKE-A). This interaction between STAT3 and PIKE-A forms a binding partnership that recruits Fyn, a protein kinase responsible for phosphorylating STAT3. As a result, this cascade leads to the upregulation of G6PD expression, promoting the development of tumors while simultaneously inhibiting cellular senescence [34]. These findings collectively suggest that there is an overall increase in the expression of glycolytic and PPP genes. This upregulation facilitates the production of additional ATP and nucleotides, which are essential for the uncontrolled proliferation of GBM cells.

### Glutamine metabolism

Glutamine plays a crucial role in providing energy and carbon sources for the proliferation of cancer cells. It is absorbed by various transporters located on the cell membrane [35]. Furthermore, glutamate is enzymatically produced from glutamine and serves as a critical substrate for the synthesis of lipids, nucleotides, and amino acids. Notably, the conversion of glutamate to glutamine is a tightly regulated process, particularly in the absence of adequate glutamine levels, primarily through the glutamine synthetase (GS) pathway. The generated glutamine plays a crucial role in promoting cell growth and supporting nucleotide biosynthesis in established GBM cell lines and astrocytes [36]. Indeed, expression of GS is dramatically high in primary GBM cells that showed cancer stem cell properties.

Numerous studies have revealed the dysregulated metabolism of glutamine in malignant tumors. Increased levels of glutamine are utilized to sustain tumor growth by diverting molecules from the TCA cycle towards alternative metabolic pathways in GBM. Additionally, glutamate can be converted into α-KG, a critical intermediate metabolite in the TCA cycle. As a result, an excess of glutamine and glucose is often observed in GBM, underscoring their role in providing supplementary energy for the highly proliferating cancer cells [37]. Wise et al. reported that GBM cells display increased glutamine uptake and metabolism, even in the presence of abundant glucose. Furthermore, they demonstrated that depriving GBM cells of glutamine significantly inhibits cell viability *in vitro* [38]. A recent study further substantiated the critical role of glutamine in supporting cell survival in various GBM cell lines. The researchers specifically examined the effects of a glutamine antagonist prodrug called JHU-083 on GBM cells. Their findings demonstrated that JHU-083 effectively suppressed the growth of GBM cells and induced significant alterations in cellular metabolism. Additionally, the study revealed that JHU-083 inhibited mTOR signaling and led to a decrease in the expression of Cyclin D1, a protein involved in regulating cell cycle progression. These findings offer valuable additional evidence supporting the therapeutic potential of targeting glutamine metabolism in GBM [39]. Approximately 50 % of GBM patients exhibit genetic alterations in the epidermal growth factor receptor (EGFR) [40]. In a recent study by Yang et al., it was discovered that activated EGFR promotes an upregulation of glutamine metabolism through a pathway dependent on glutamate dehydrogenase 1 (GDH1). Notably, the knockdown of GDH1 resulted in a significant reduction in GBM cell proliferation and tumorigenesis [41]. By targeting these aspects of glutamine metabolism, it may be possible to develop effective therapeutic approaches for GBM. Consequently, interventions that aim to manipulate glutamine metabolism represent promising treatment strategies for GBM. Potential therapeutic approaches may involve suppressing glutamine uptake, regulating the activity of enzymes involved in glutamine metabolism such as glutamate dehydrogenase and glutamine aminohydrolase, targeting glutamate transport, and counteracting the effects of lactate.

### Lactate metabolism and acidosis

Excessive lactate production is a consequence of increased glycolysis in cancer cells. Tumor tissues exhibit lactate levels nearly 20 times higher than those in normal tissue [42]. This leads to the accumulation and secretion of acidic metabolites by monocarboxylate transporters (MCTs), resulting in the acidification of TME [43]. Moreover, lactate plays a crucial role in driving tumorigenesis, metastasis, and immune invasion [44].

Lactic acidosis is frequently observed in malignant tumors, including GBM, and it triggers a cascade of biochemical reactions that alter metabolism and signaling pathways. Most tumors exhibit enhanced glycolysis and defective OXPHOS. This altered metabolism promotes tumor cell growth and enables them to resist adverse microenvironments in a Warburg effect-dependent manner, ultimately leading to an excessive production of lactate [45]. Furthermore, lactic acidosis also contributes to drug resistance and immune escape in GBM. Microglia is considered one of the most crucial immune cells in the central nervous system and GBM immunology, playing a significant role in tumorigenesis. Moreover, the expression of insulin-like Growth Factor Binding Protein 6 (IGFBP6) contributes to immune evasion, migration, and inflammation in GBM. Lonhitano et al. demonstrated that lactate induces the expression of MCT1 and IGFBP6 in microglia cells, indicating a crosstalk between lactate and IGFBP6. The GBM zebrafish animal model and analysis of transcriptome datasets from human GBM biopsies confirmed that lactate regulates IGFBP6 expression in GBM cells. This, in turn, modulates microglia polarization to promote tumor progression and resistance to therapy. Additionally, they found that lactate can maintain high ATP levels and prevent cell death. Furthermore, *in vivo* studies have provided confirmation that the inhibition of MCTs function in GBM leads to impaired glycolysis [46,47]. Another study has demonstrated that lactate promotes the growth and progression of GBM cells by increasing the expression of MCT1 and its receptor, hydroxycarboxylic acid receptor 1 (HCAR1) [48]. Pyruvate is a crucial intermediate metabolite involved in the formation of lactate and the TCA cycle. Mitochondrial pyruvate carriers (MPCs) play a pivotal role in translocating pyruvate from the cytosol to the mitochondria, which is closely associated with tumor metabolism and biosynthesis processes. Chai et al. performed an analysis of genomic and clinical data from 631 GBM patients in The Cancer Genome Atlas (TCGA) and discovered that the deletion of MPC1 is correlated with a poorer prognosis and resistance to temozolomide(TMZ) in GBM [49,50]. Not only does lactate significantly enhance the proliferation, migration, and colony formation capacity of GBM cells, but it also has an impact on the expression of epithelial-mesenchymal transition (EMT) protein markers, including E-cadherin and β-catenin [51].

### Fatty acid metabolism

Lipids play a critical role in brain structure and function, particularly in cell membrane integrity and biosynthesis of specific proteins in CNS. The brain's reliance on lipids extends beyond their role in membrane integrity and protein biosynthesis. Lipids also serve as a vital source of energy for various brain functions, including neurotransmission and synaptic plasticity [51], [52], [53]. As it is well established, dysregulations in fatty acid (FA) metabolism, including upregulated FA biosynthesis, accumulation of fat droplets for energy storage, and increased catabolism, have been found to contribute to tumorigenesis, disease progression, and therapy resistance in cancer [54]. Abnormal accumulation of lipid droplets has been observed in both GBM cell lines and GBM patients. This aberrant accumulation of FA metabolites has been associated with a lower survival rate in GBM patients [55]. Studies have reported that the most abundant FA found in GBM are palmitic acid and oleic acid [56]. Furthermore, dysregulation of fatty acid (FA) metabolism has been implicated in promoting inflammation in GBM. Arachidonic acid, a polyunsaturated fatty acid (PUFA), serves as a precursor for a family of bioactive molecules involved in inflammation, including prostaglandins and leukotrienes. Nicolaou et al. observed significant correlations between poor patient survival and high expression levels of microsomal PGE synthase 1 and prostaglandin reductase 1 mRNA. These enzymes are involved in the synthesis of prostaglandins, highlighting their potential role in GBM inflammation [57]. Elevated levels of FA can promote the proliferation of cancer cells when these metastatic cells migrate across the blood-brain barrier (BBB) into the brain parenchyma. A previous study discovered that PUFAs released by inflammation-activated astrocytes serve as a source for metastatic cancer cells to form cell membranes. However, a contrasting study demonstrated that omega-3 induced GBM cell death and enhanced the effects of radiotherapy both *in vitro* and *in vivo* [58,59].

Cholesterol, a crucial lipid molecule for cells, plays a vital role in various biological processes as it serves as an essential component of cell membranes and is involved in the production of metabolites. Recent research has revealed that elevated levels of cholesterol contribute to increased tumorigenesis and metastasis in cells [57,59]. Moreover, the survival of GBM cells is dependent on cholesterol. Villa et al. discovered that GBM cells exhibit an increased uptake of cholesterol and upregulate low-density lipoprotein (LDL) receptors, suppressing the synthesis of endogenous cholesterol and oxysterols. These alterations enable GBM cells to evade feedback mechanisms and disrupt the balance of cholesterol homeostasis [60].

Low tumor oxygenation and hypoxia are characteristic features of GBM that contribute to cancer cell invasion, drug resistance, and suppression of antitumor immune responses. Additionally, hypoxia promotes the uptake of FAs by fatty acid-binding protein 3 and 7 (FABP3 and FABP7) in GBM These FAs can be stored in lipid droplets and serve as a potential energy source to support the survival of GBM cells during hypoxia-reoxygenation cycles [61]. Fatty acid β-oxidation (FAO) has been identified as a prominent metabolic pathway in GBM through comprehensive analysis that combines global metabolomic and gene expression profiling on samples derived from GBM patients. This integrative approach has revealed that fatty acid β-oxidation plays a crucial role in GBM metabolism, highlighting its significance as a dominant metabolic node in the disease. Enhanced FAO enables GBM cells to adapt to the dynamic TME [61,62].

**Fig. 1:**
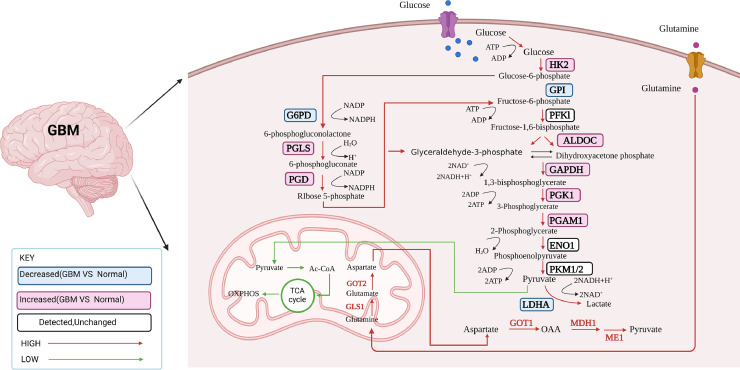
Shows a summary of the metabolic adjustments in GBM. A streamlined model demonstrating how the genes for metabolic enzymes change as GBM develops the rise in genes linked to rate-limiting enzymes in glycolysis, including HK2, ALDOC, GAPDH, PGK1, and PGAM1, is one of the most obvious transcriptional alterations throughout the development of GBM. This enables the conversion of glucose to pyruvate upon entry into the cells. The PPP-associated genes are also increasing, indicating that PPP has entered its oxidative phase. Surprisingly, GBM has downregulated the TCA cycle and OXPHOS genes, indicating that glycolysis is the primary source of energy for GBM. Biorender was used to construct the figure.

Tumor development relies on the rewiring of cellular metabolism, which involves the ability of tumor cells to extract essential nutrients from nutrient-depleted environments and utilize them to sustain cell viability and generate new cellular components. GBM exhibits significant metabolic alterations, including increased fatty acid uptake and oxidation, enhanced cholesterol metabolism, and a reliance on glucose metabolism. These metabolic changes support the high energy demands of GBM cells and contribute to tumor growth, invasion, and resistance to therapy. Additionally, GBM cells display a preference for glutamine metabolism, which fuels biosynthetic pathways and supports cell proliferation. Understanding metabolic reprogramming in GBM presents potential targets for therapeutic interventions and underscores the importance of considering metabolic pathways in the development of novel treatment strategies (Fig. 1).

## Signaling networks of metabolic reprogramming

### PI3K/AKT/mTOR driving anabolic metabolism

The PI3K-Akt-mTOR pathway is among the most commonly altered pathways in various types of tumors. A multitude of studies have demonstrated that activation of this pathway promotes proliferation in cancer cells. Oncogene signaling pathways are closely related to reprogrammed metabolism in cancers. A previous study demonstrated that the activation of the PI3K/Akt pathway induces glucose uptake [63]. Additionally, Akt or PI3K can function as effectors that regulate downstream signaling pathways through phosphoinositide phosphatases and other related metabolites [64,65]. Through the regulation of cellular REDOX processes, nucleotide metabolism, lipid synthesis, and protein synthesis, the activation of the PI3K/AKT/mTOR pathway facilitates the direct fulfillment of energy requirements in rapidly growing tumor cells. This metabolic reprogramming is accomplished by the direct control of numerous crucial processes involved in glycolysis and the TCA cycle. Specifically, the activation of this pathway influences the activities of key enzymes such as HK2, ATP-citrate lyase (ACL), and the stabilization of hypoxia-inducible factor 1-alpha (HIF1a), ultimately enabling the tumor cells to meet their energy needs [66]. Analysis of primary GBM patients has revealed the activation of AKT, mTOR, forkhead box O transcription factors, and S6 activation [67]. This finding is further supported by Li et al., who reported significantly higher levels of phosphorylation of AKT, mTOR, and S6K in high-grade gliomas compared to low-grade gliomas [68].

Activation of Akt not only directly promotes the activity of GBM cells, but also serves as a critical mediator that affects numerous transcription factors and metabolic enzymes in cancer. HK2 phosphorylates glucose, converting it into G-6-P, which is a critical metabolite involved in various pathways such as glycolysis, PPP, hexosamine biosynthesis, ATP synthesis, and glucose storage. The activation of HK2 depends on the phosphorylation of AKT. Moreover, under specific conditions, AKT inhibits the FOXO family of transcription factors, which promotes glycolysis by maintaining the suppression of MYC. The MYC oncogenes encode a family of transcription factors that play a crucial role in regulating cell cycle proteins [69]. Additionally, AKT triggers the expression and membrane translocation of GLUT to affect the glycolytic phenotype of GBM [70].

As a downstream target of the PI3K/AKT pathway, mTOR is a central regulator in cancer metabolism that promotes the biosynthetic demands necessary for GBM survival. It is also associated with the induction of the Warburg effect by inducing the expression of glucose transporter type 4 (GLUT4) and activating HK2 and PFK-1 [71]. Numerous studies have demonstrated that activation of the mTOR signaling pathway leads to the production of transcription factors that subsequently increase the expression of glycolytic genes. Additionally, the PI3K/AKT/mTOR pathway enhances the uptake of glucose. Shreya et al. found that inhibiting PI3K-mTOR resulted in decreased levels of glycolytic metabolites, including a significant reduction in NAD+ and glutamate metabolites in GBM. Moreover, decreased glucose uptake and lactate secretion were also observed in this study [72].

In summary, the PI3K-AKT-mTOR signaling pathway plays a vital role in the development and progression of GBM. The activation of this pathway contributes to cell proliferation, survival, invasion, and metastasis, as well as being linked to tumor angiogenesis and treatment resistance in GBM. Consequently, targeting this pathway has emerged as a key research focus for GBM treatment, with inhibitors demonstrating significant potential.

### Hypoxia/HIFs

**Fig. 2:**
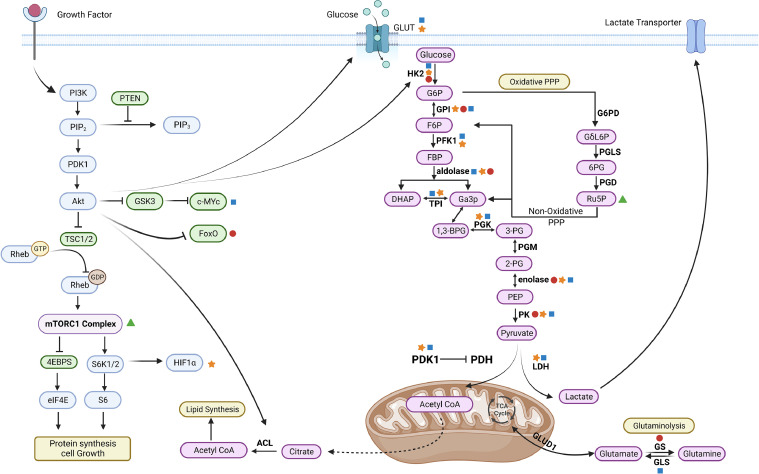
Shows the benefits of Warburg and molecular signaling. Glycolysis and the tricarboxylic acid (TCA) cycle are directly controlled at a number of stages by the PI3K-AKT-mTOR signaling pathway, which is significantly dysregulated in glioblastoma. By encouraging increases in glucose transport and hexokinase activity (HK2), AKT may facilitate aerobic glycolysis. ACL-dependent conversion of citrate to cytosolic acetyl-CoA for fatty acid synthesis may also be encouraged by increased AKT activity. Increased glycolysis may encourage nucleotide synthesis and provide equivalents for REDOX that can reduce NADH. Glycolysis may be slowed down by inactive PKM2 by shifting intermediate metabolites to anabolic pathways. Pyruvate production may be switched from lactate to lactate instead of pyruvate if HIF1a is stabilized by mTORC1. Biorender was used to construct the figure.

Intratumoral hypoxia is a characteristic feature of GBM and is associated with resistance to therapy, immune evasion, and the maintenance of cancer stem cells [73,74]. Hypoxia-inducible factor-1 (HIF-1), a crucial regulator of cellular response to hypoxia, plays a significant role in promoting cell growth, invasion, genetic alterations, and metabolic reprogramming in various types of tumors [75,76]. HIF-1 is also implicated in the initiation and progression of GBM. Activation of HIF-1 leads to the upregulation of numerous genes involved in metabolism, such as glucose transporters and enzymes in glycolysis. This results in a metabolic shift from OXPHOS to aerobic glycolysis, also known as the Warburg effect, in response to limited oxygen availability [77,78]. Furthermore, HIF-1 directly increases the expression of phosphoinositide-dependent kinase 1 (PDK1), which promotes lactate production and lowers the surrounding pH [79]. The presence of lactate and an acidic microenvironment not only supports tumor growth but also reduces the effectiveness of many anti-tumor drugs [80]. Additionally, HIF-1 can induce the overexpression of drug transporters, leading to the efflux of drugs from tumor cells [81]. The activation of glycolysis also results in the synthesis of excessive ATP, which serves as an energy source and contributes to drug resistance mediated by HIF-1. Therefore, targeting HIF-1 and its associated metabolic pathways holds potential as a therapeutic strategy in cancer therapy (Fig. 2).

## Treatment opportunity: targeting the metabolic dependence of glioblastoma

As we describe above, GBM is known to exhibit a unique metabolic profile characterized by increased glucose uptake and reliance on aerobic glycolysis, also known as the Warburg effect. Targeting the altered metabolism of GBM cells has gained attention as a therapeutic strategy. Several approaches have been explored, including the inhibition of glucose uptake, which is considered a direct and effective method to reverse tumor metabolism. The glucose analog 2-deoxy-glucose (2-DG) inhibits the phosphorylation of glucose by hexokinase, effectively blocking glucose metabolism in tumor cells. Beata et al, demonstrated that WP1234, a compound capable of releasing 2-DG inside the cells through metabolism, showed promising results as a novel anticancer agent in a GBM model [82].

Hyperactive glycolysis is an important characteristic of many malignant tumors. Targeting glycolysis is also a possible strategy in tumor treatment. Dimethylaminomicheliolide (DMAMCL) is a small molecular compound that alters glycolysis and decreases the proliferation of GBM cells through the activation of PKM2. DMAMCL has been used in clinical trials for recurrent GBM [83]. Recent research has suggested that enzymes involved in metabolic pathways could serve as potential targets for cancer treatment. One such compound is Devimistat, also known as CPI-613, which specifically targets enzymes involved in the energy metabolism of cancer cells, including pyruvate dehydrogenase and alpha-ketoglutarate dehydrogenase. In preclinical studies using a GBM animal model, CPI-613 has shown promising results. It effectively reduces the levels of metabolites in the TCA cycle which leads to altered energy metabolism and ultimately decreases cancer cell proliferation. Furthermore, CPI-613 has been shown to prolong the overall survival time in the GBM animal model [84].

Mitochondria are indeed a potential target for cancer treatment, including in the case of glioblastoma (GBM). Metformin, a commonly used anti-diabetic drug, has been found to have potential anti-cancer effects by acting as an OXPHOS inhibitor to induce cell death in GBM. Metformin's ability to inhibit OXPHOS disrupts the mitochondrial energy production process, leading to a decrease in ATP production and an increase in cellular stress. This can ultimately result in cell death in GBM cells [85]. Another OXPHOS inhibitor, Gboxin, has been found to specifically inhibit the growth of primary GBM cells. Gboxin targets the mitochondrial complex I, disrupting the OXPHOS process and leading to reduced ATP production and increased cellular stress in GBM cells [86]. Several studies have demonstrated the potent suppression of the oxygen consumption rate in various tumor cells, including GBM, by gamitrinib, which is a mitochondrial matrix inhibitor known as geldanamycin (GA). Currently, gamitrinib is undergoing assessment in a phase I clinical trial involving patients with advanced malignancies. The phase I trial will provide crucial insights into the tolerability and effectiveness of gamitrinib in treating advanced malignancies, including GBM. Further research and clinical trials are necessary to determine the full potential of gamitrinib as a therapeutic option for cancer patients [87], [88], [89].

**Fig. 3:**
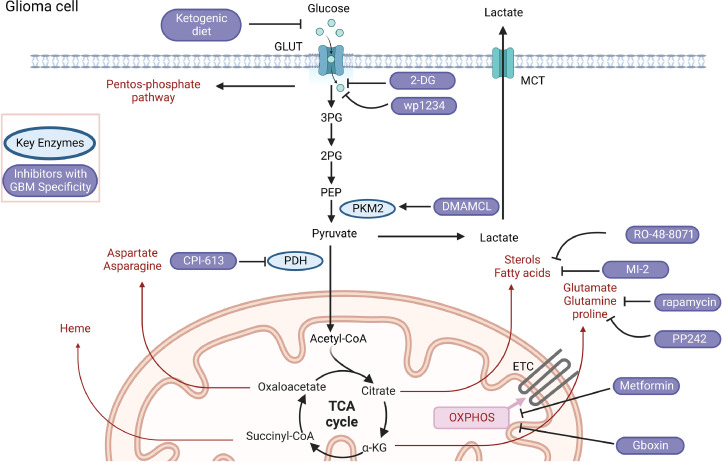
Strategies to Prevent GBM Cell Metabolism. Therapeutic strategies for targeting the abnormal metabolism of GBM starting cells are being investigated. Biorender was used to construct the figure.

The mTOR pathway is a crucial regulator of the PI3K/AKT pathway and is considered a therapeutic target in various types of tumors, including GBM. Targeting mTOR has been shown to impact glutamine metabolism, leading to the suppression of cell proliferation, glucose uptake, and lactate production in GBM. Furthermore, dysregulation of lipid metabolism is a prominent metabolic alteration observed in cancer cells. Targeting lipid metabolites has emerged as a potential therapeutic strategy. Pharmacological inhibitors of lanosterol synthase, such as MI-1 and RO-48-8071, have been used to selectively kill H3-K27M-mutant diffuse intrinsic pontine glioma and GBM cells. These inhibitors have also been found to increase the production of endogenous liver X receptor (LXR) ligands. This suggests that targeting the LXR-cholesterol axis may present an actionable vulnerability in multiple glioma subtypes. These findings highlight the potential of targeting the mTOR pathway and lipid metabolism as therapeutic strategies for GBM. However, further research and clinical trials are needed to fully understand the effectiveness and safety of these approaches in treating GBM patients [33](Fig. 3).

## Conclusions

GBM, the most prevalent and aggressive malignant brain tumor in adults, poses significant challenges due to its heterogeneity and complex TME, leading to poor prognosis and limited treatment options. Numerous studies have explored different pathways and target genes that undergo alterations in GBM. However, despite these efforts, there have been limited advancements in improving patient survival or quality of life. Metabolomics, a rapidly evolving field, offers a promising avenue to identify the molecular pathways that underlie the functionality of GBM. Recent advancements in metabolomics analysis have shed light on the intricate metabolic reprogramming and underlying molecular mechanisms of GBM. As our comprehension of the diverse mechanisms and distinct metabolic profiles of GBM expands, it becomes increasingly feasible to develop innovative therapeutic interventions customized to individual patients, taking into account their specific genetic and phenotypic characteristics. This article aims to provide a comprehensive overview of the metabolic foundation of GBM, with the ultimate goal of enhancing disease outcomes by unraveling the mechanisms of tumor metabolism and identifying potential therapeutic targets.

## Funding

This work was supported by Sponsored by 10.13039/501100005090Beijing Nova Program (20220484218).

## CRediT authorship contribution statement

**Jinyi Zhao:** Writing – review & editing. **Xuemei Ma:** Writing – review & editing. **Peixian Gao:** Writing – review & editing. **Xueqi Han:** Writing – review & editing. **Pengxiang Zhao:** Writing – review & editing. **Fei Xie:** Writing – review & editing. **Mengyu Liu:** Conceptualization, Writing – review & editing.

## Declaration of competing interest

The authors declare that the research was conducted in the absence of any commercial or financial relationships that could be construed as a potential conflict of interest.
